# Atypical clinical and sonographic manifestations of lymphadenopathy in a child with cat-scratch disease: A case report

**DOI:** 10.1007/s40477-024-00923-7

**Published:** 2024-06-22

**Authors:** Laura Bruni, Michelangelo Baldazzi, Laura Greco, Donatella Vivacqua, Anna Olga Di Vincenzo, Ilaria Corsini, Stefano Bruni, Rocco Minelli, Eugenio Rossi, Giuseppe Paviglianiti, Marcello Napolitano, Marcello Lanari, Luigi Lovato

**Affiliations:** 1 Alma Mater Studiorum-Università di Bologna, IRCCS Azienda Ospedaliero-Universitaria di Bologna, Bologna, Italy; 2 Pediatric and Adult CardioThoracic and Vascular, Oncohematologic and Emergency Radiology Unit, IRCCS Azienda Ospedaliero-Universitaria di Bologna, Bologna, Italy; 3 Unit of Paediatric Emergency, Department of Medical and Surgical Sciences, Sant’Orsola-Malpighi University Hospital, Bologna, Italy; 4 Bologna, Italy; 5 Department of Medicine and Health Sciences “Vincenzo Tiberio”, University of Molise, Via Francesco De Sanctis, 1, 86100 Campobasso, CB Italy; 6 U.O.S.D. Diagnostica per Immagini A.O.R.N. Santobono-Pausilipon, Pausilipon Hospital, Naples, Italy; 7 UOC Radiologia Pediatrica ARNAS Civico-Di Cristina-Benfratelli, Palermo, Italy; 8 Department of Paediatric Radiology and Neuroradiology, V. Buzzi Children’s Hospital, 32 Castelvetro St., 20154 Milan, Italy

**Keywords:** Cat scratch disease, Pediatric, Lymphadenopathy, Ultrasound

## Abstract

Cat-scratch disease is a well-known infection in childhood. It usually presents as tender lymphadenopathy and should be included in the differential diagnosis of any lymphadenopathy syndrome. An history of exposure to cats supports the suspect and a positive serologic test to *Bartonella henselae* confirms the diagnosis. Ultrasound is the first line radiologic imaging performed in case of lymphadenopathy. The presence of hypoechoic lobular or oval mass with central hyperaemia and a possible adjacent fluid collection and surrounding oedema may differentiate the disease from other aetiologies. We describe the case of a 7-year-old girl presenting with an axillary lymphadenopathy, without a reported recent history of exposure to cats, with sonographic findings suggestive for cat-scratch disease. In this case, ultrasound was very useful in orienteering the diagnosis and insist on the medical history. Serology resulted positive for *B. henselae* and at the end the family remembered that 6 months before the child was scratched by a kitten.

## Introduction

Cat-scratch disease is a well known infection in childhood. It is transmitted through a cat scratch or bite and usually presents as regional lymphadenopathy, in most cases affecting the upper limb. General symptoms are usually mild and flu-like. In the majority of cases the disease is self-limiting and does not require antibiotic therapy. However a disseminated form, characterised by systemic manifestations and sometimes serious complications, is also possible [1, 2].

The disease should be included in the differential diagnosis of any lymphadenopathy syndrome [3]. History of exposure to cats supports the suspect and positive serologic test to *Bartonella henselae* confirms the diagnosis.

In the regional form, the sonographic finding is that of a hypoechoic lobular or oval mass with central hyperemia and a possible adjacent fluid collection [4–9]. When the necrosis becomes evident and alters the normal structure of the lymph node, the lesion may suggest a soft tissue tumor [10].

The regional form usually resolves spontaneously within few weeks [11]. Anyway, the use of specific antibiotic therapy aims at preventing serious complications, such as dissemination to the liver, spleen, eye, or central nervous system (which happens in 14% of non-treated patients). In addition, data suggest that antibiotic treatment can shorten the duration of symptoms. A 5-day course of azithromycin is considered the first choice.

## Case Report

**Fig. 1:**
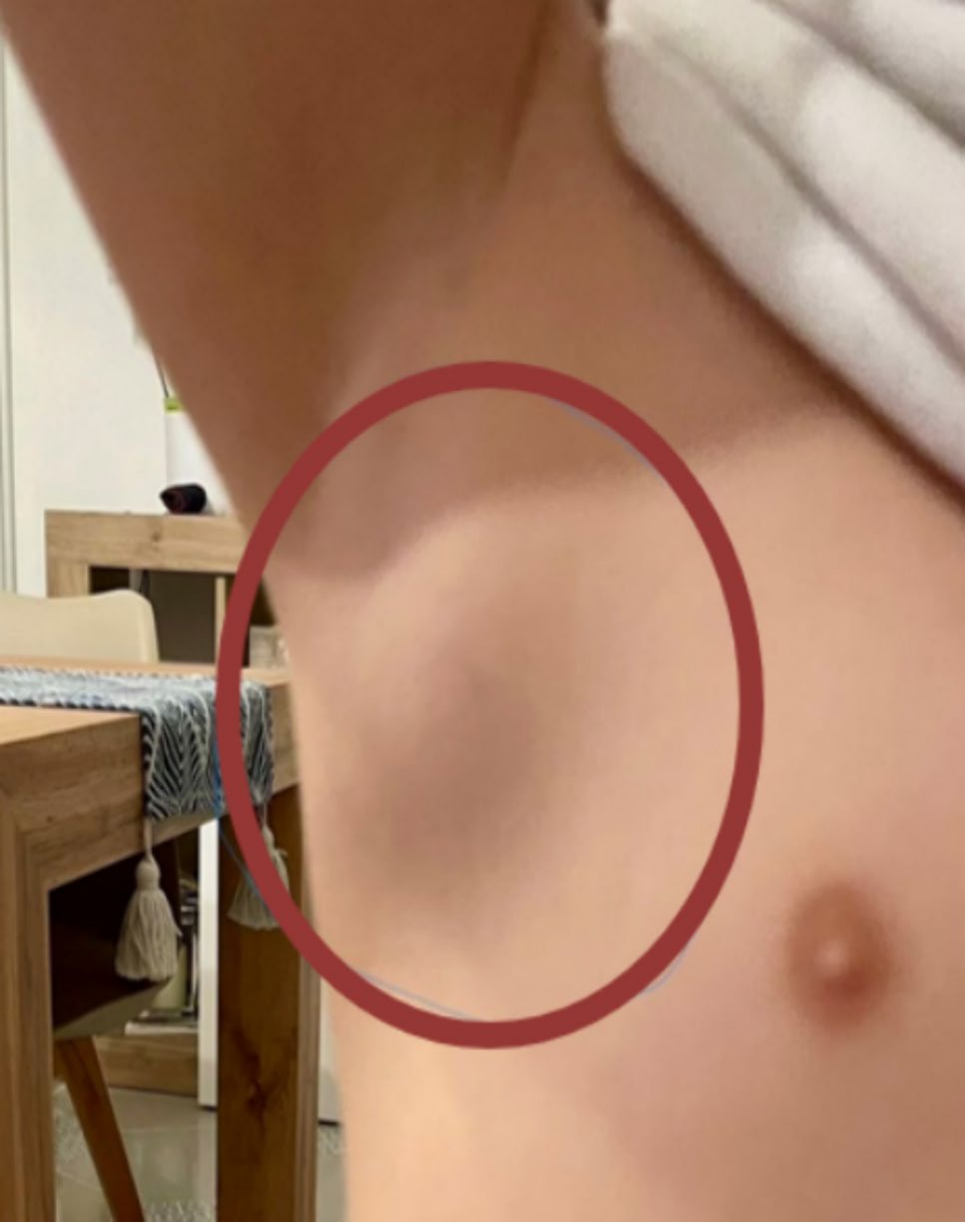
Clinical presentation

We describe the case of a 7-year-old girl, presenting to her general practitioner with an history of 8-days-lasting low-grade fever and the concomitant presence of a painful, erythematous swelling in the right axillary cavity progressively increasing in size (Fig. 1).

At a first interview, her family and personal history was apparently silent. No travels nor contacts with animals of any kind were mentioned. In particular, no exposure to cats nor tick bite was reported at the beginning.

**Fig. 2:**
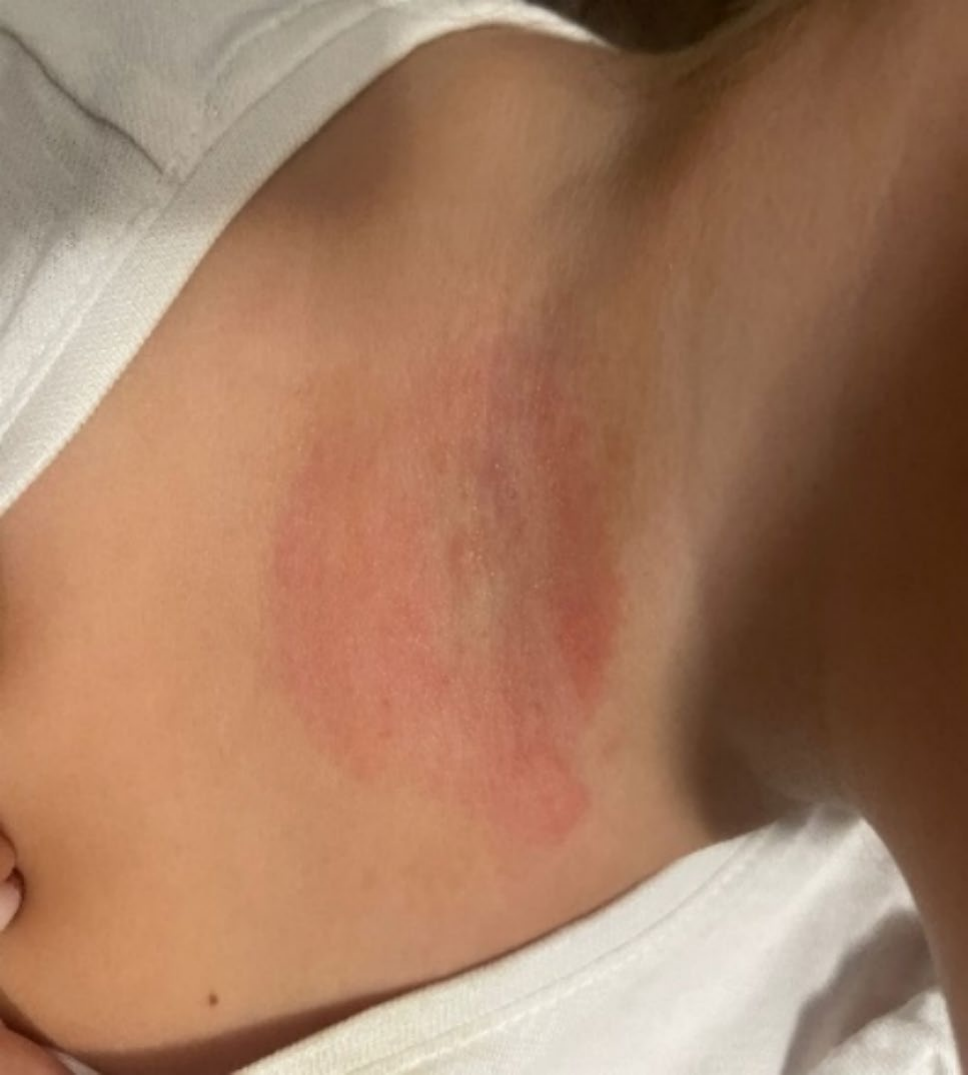
Anamnestic confounding lesion

Since months earlier, after playing in a park, the little girl had been stung on the hand and her neck by an unidentified insect and following these bites the girl had shown a slowly resolving rash at the base of her neck (Fig. 2), the family pediatrician had carried out some blood tests. They showed a leukocytosis (13.890/mmc), neutrophilic (9.220/mmc) and high inflammatory markers (ESR 83 mm, nv < 17 and CRP 2.80 mg/dl, nv < 0.5). Serological test for *Ebstein Barr virus* and *Citomegalovirus* was negative; IgG to *Borrelia burgdoferi* borderline (IgM negative). Due to the evidence of a high Antistreptolysin-O titer (1091 UI/ml, nv < 200) and anti-dnase b streptococcal antibodies (3720 UI/ml, nv < 200), the girl underwent a cycle of 10 days antibiotic therapy with Amoxicillin. Fever had disappeared but no reduction in the size of the axillary swelling was noted.

For this reason, an ultrasound in a private clinic was performed, showing a bi-lobed oval formation referable to a lymph node increased in volume (25 mm), with an increased and diffuse vascular pattern with loss of the normal vascular pole. Urgent needle aspiration was recommended.

**Fig. 3:**
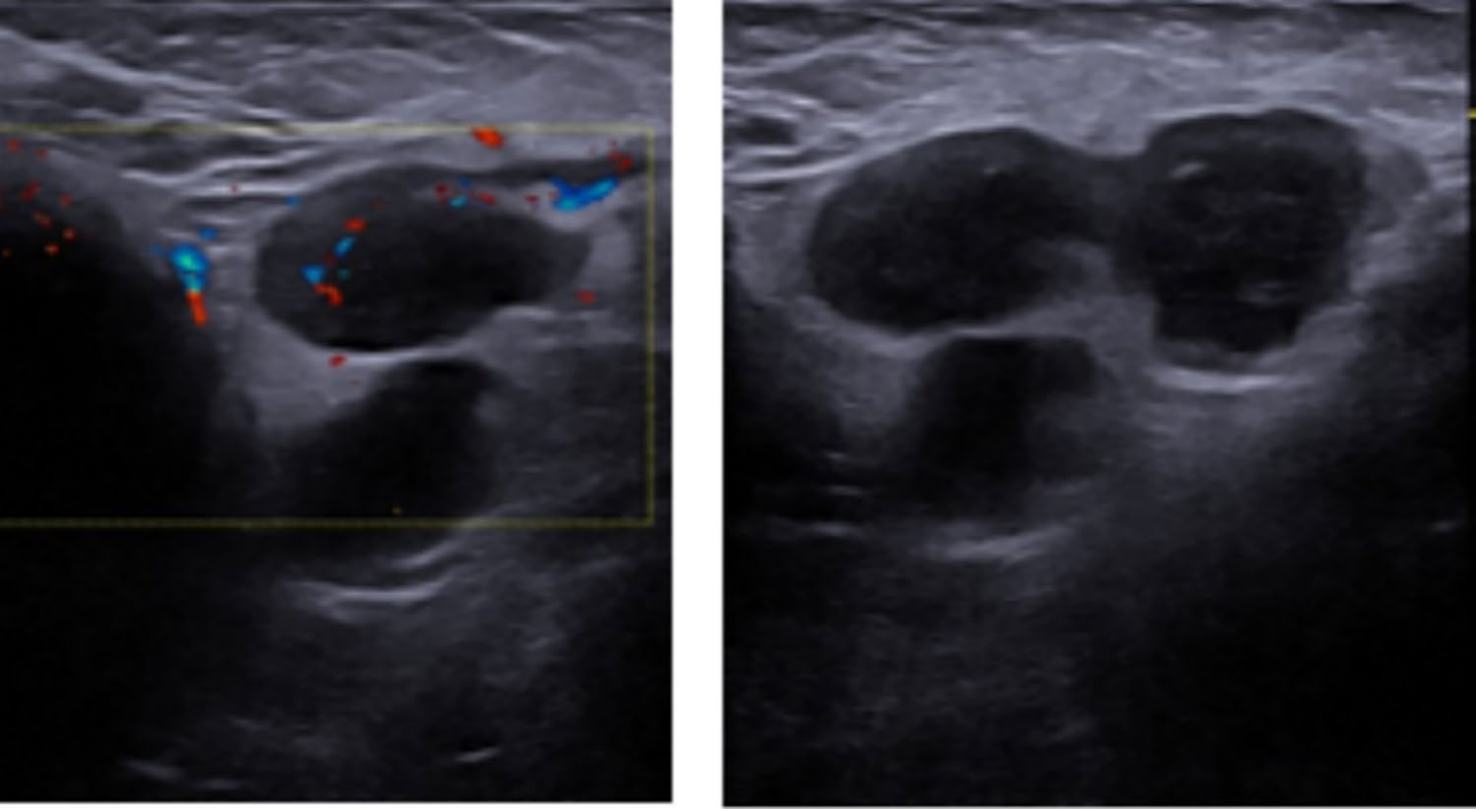
Ultrasound analysis at presentation

Before proceeding with the invasive needle aspiration, a second opinion was requested, and another ultrasound was performed at our Pediatric Radiologic Center (Fig. 3). It showed, at the level of the clinical finding in the axillary subcutaneous tissues, 4–5 roundish lesions with marked hypoechogenicity. The diameter of every single lesion was 15–20 mm. They were all close to each other and tending to coalescence. They all had intra-lesion Doppler signal without aspects of hyperemia and a vascular hilum wasn’t recognizable. A peculiarity was that the lesions described were connected to each other by the presence of thin hypoechoic bridges. The soft tissues all around the ultrasound finding were hyperechoic. The exam was extended to the other axillary cavity, abdomen and neck without relevant findings (only later cervical lymph nodes with reactive characteristics were noticed).

In the suspect of a lymphadenitis with an important alteration of lymph nodes eco structure, another cycle of antibiotic (Amoxicillin-Clavulanic acid and Azithromycin for 7 days) was prescribed. At the follow-up examination one week later, a clinical and ultrasound worsening of the lesion was described: it was tending more to coalescence, areas of necrosis appeared and the aspects of edema and inflammation of the locoregional soft tissues were more evident. In particular, the colliquated material, which had exited the lymph nodes, had spread into the neighboring tissues.

**Fig. 4:**
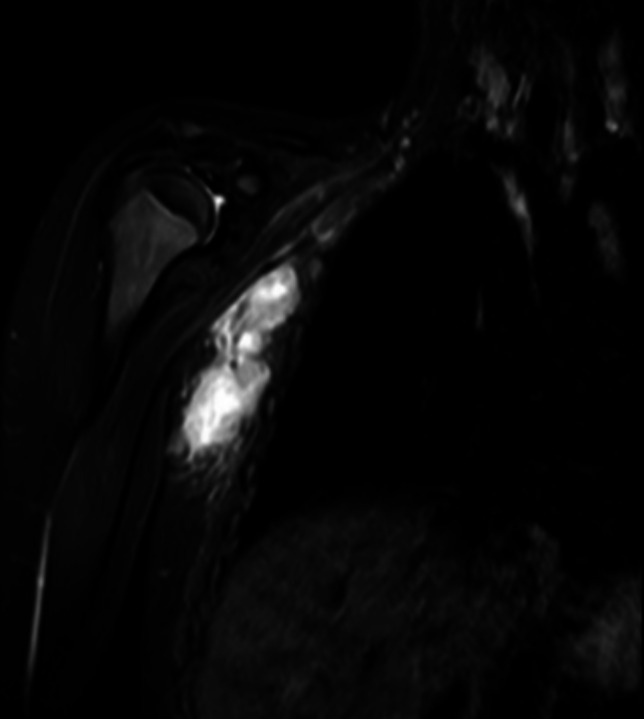
MRI imaging confirms the presence of multiple lymphadenopathy without malignant signs

To characterize the lesion with a better resolution and a more panoramic view and to study its extension in depth, an MRI was performed (Fig. 4). The exam confirmed the presence of many adenopathies in the right axillary cavity, at all levels of the subcutaneous tissues. The dimension ranged from few mm to 3 cm. Those of greater dimension showed nuanced aspect of hypodense suggestive for colliquative necrosis.

In the increasingly strong suspicion of an infectious disease, further serological tests were carried out: anti *Toxoplasma Gondii* and anti *B. burgdoferi* antibodies negative, Quantiferon TB-plus negative; intradermal Mantoux reaction negative; IgG and IgM positive for *B. henselae*.

Investigating more in depth the medical history, it emerged that the girl was scratched by a kitten 6 months before. Following this finding and given the persistence of altered inflammation markers, the child underwent a further cycle of Azytromicin, the hypothesis of access to the operating room for surgical excision of the lesion was set aside and a clinical and ultrasound follow up was performed.

**Fig. 5:**
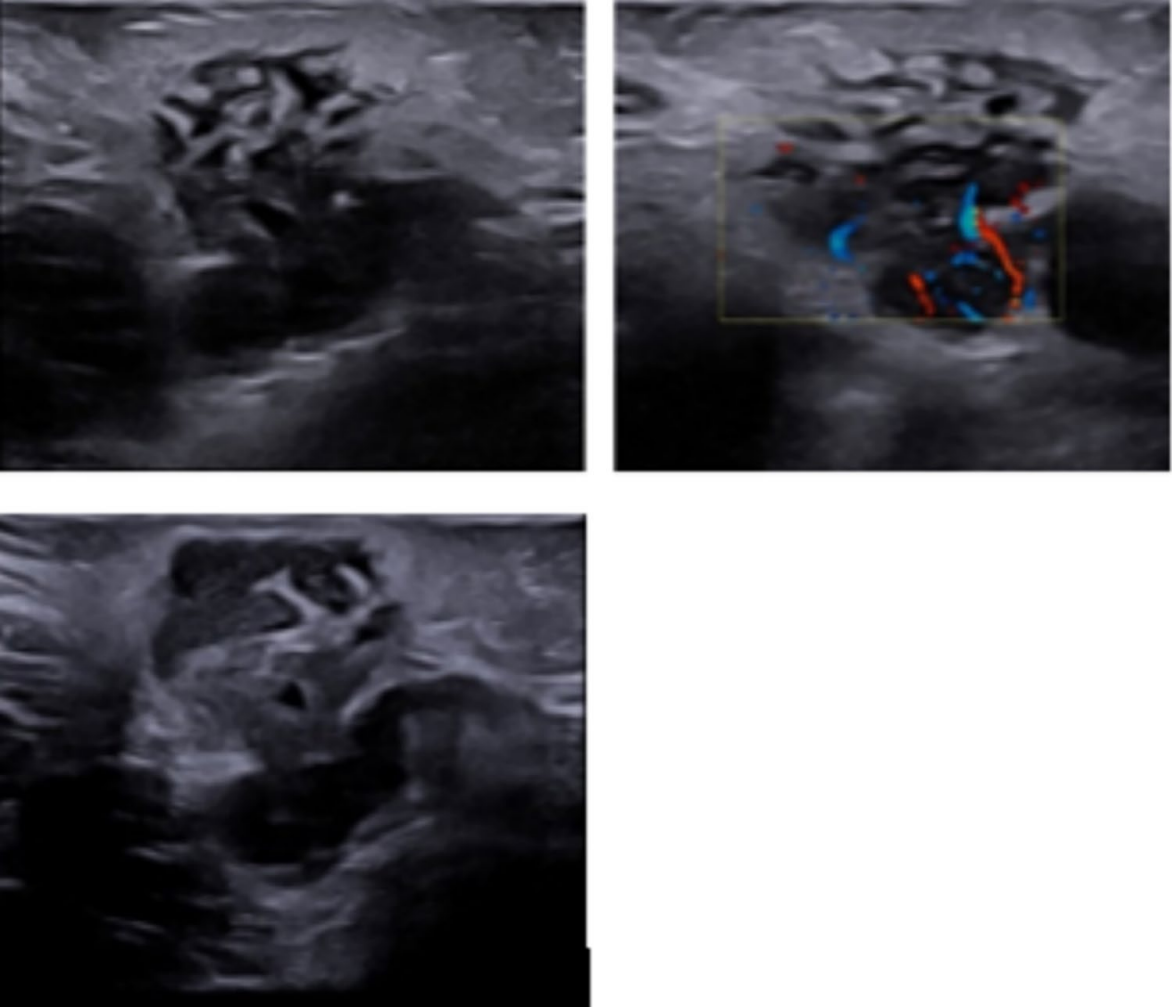
Follow-up 2 months after the disease onset: lymph nodes with reactive characteristics in contact with an inhomogeneous area without Doppler signal referable to necrotic-colliquative cicatricial outcomes; gradual resolution of the inflammation of the soft tissues around the lesion

At the last follow up (2 month after the disease onset) the clinical examination showed a 2 cm small adenopathy, no longer painful nor erythematous. The ultrasound revealed three lymph nodes with reactive characteristics, the larger of which with a diameter of 21 × 6 mm (Fig 5). The more superficial was in contact with an inhomogeneous area without Doppler signal referable to necrotic-colliquative cicatricial outcomes. The inflammation of the soft tissues around the lesion resolved.

## Discussion

Cat-scratch disease is a bacterial infection that frequently affects the child [1]. In the localised form of the disease, enlarged lymph nodes appear proximal to the inoculation site, about two weeks (range 7–60 days) after the *B. henselae* is inoculated into the skin through a cat’s scratch or bite. The diagnosis is suspected in the presence of axillary or cervical lymphadenopathy with a history of cat’s exposure. Even if the regional form usually resolves spontaneously within few weeks, a short course of azithromycin is suggested to prevent dissemination of the disease to abdominal organs and/or central nervous system and to shorten the duration of symptoms [11].

The one we described is a case of a localized lymphadenitis in cat-scratch disease. We want to underline some clinical and sonographic peculiarities.

From a clinical point of view our case differs from most of the previously described ones, since the onset of the disease was 6 months after the contact with a kitten, quite a long time compared to the incubation period described in literature (about 2 weeks). This is maybe the reason why parents had forgotten the episode and, when asked, no exposure to cats was mentioned at the beginning. Only with the increasing clinical suspicion, investigating more in depth the medical history, they finally remembered. The second peculiarity is that the disease didn’t respond clinically nor echographically to the first cycle of antibiotic (Azithromycin combined with Amoxicillin-Clavulanic acid); on the contrary, it worsened, and a second cycle of Azithromycin was necessary. Finally, the course of the disease from its onset to its resolution, despite the antibiotic therapy, was very slow. In fact, 4 months passed from the first clinical examination until the complete disappearance of the clinical and sonographic findings at the last follow-up.

From a sonographic point of view, the finding was that of roundish masses with marked hypoechogenicity tending to coalescence. They all had intra-lesion Doppler signal without aspects of hyperemia and a vascular hilum wasn’t recognizable. Apart from the abundant adjacent fluid collection, probably due to colliquated material spreading from the lymph nodes to the contiguous subcutaneous tissues, a peculiarity was that the lesions described were connected to each other by the presence of thin hypoechoic bridges. The soft tissues all around the ultrasound finding were hyperechoic.

## Conclusions

Cat-scratch disease is a quite common infection in childhood. It usually presents as tender lymphadenopathy and should be included in the differential diagnosis of any lymphadenopathy syndrome. Ultrasound is confirmed to be an excellent test for orienting clinical suspicion and, once the diagnosis has been reached, for monitoring the evolution of the disease.

## Data Availability

Data sharing is not applicable to this article, as no datasets were generated or analyzed during the current study
